# Supplementary material for: In-vitro accuracy of casts for orthodontic purposes obtained by a conventional and by a printer workflow
Source: PLoS One. 2023 Mar 15;18(3):e0282840. doi: 10.1371/journal.pone.0282840 (PMC10016668; doi:10.1371/journal.pone.0282840)
Supplement: Attachment — Submitted filename: Plos One.docx [file pone.0282840.s001.docx]

Accuracy of conventional and printed casts for orthodontic purposes

**General**

- This is a well written report.
- The study was thoroughly carried out.

**TITLE**

Adequate

**ABSTRACT**

Adequate

**INTRODUCTION**

Adequate

**MATERIALS AND METHODS**

***Materials & Instruments:***

1. Clarify if the reference cast was scanned and digitized 5 times (line no.89) or 10 times (line no.186).
2. Please describe further details of the standardization of application of artificial saliva (especially in relation to the amount) before taking alginate impression (line no. 104).
3. As described in Discussion (line no.) which states the moisture sensitive property of alginate, report on the time taken after impression until casting and whether moisture loss from alginate impression was minimized similar to normal clinical situation.

**RESULTS**

4. Suggest reporting on precision analysis results of the high-resolution scans of the reference cast [REF] (line no.185).

**DISCUSSION**

5. Discuss the widening of the arch in the molar area with intraoral scanning.

**LIMITATIONS**

6. Limitations need to be addressed, comment on inherent weakness of in vitro studying, focusing on possible differences in clinical situation. For example, intraoral scanning of the reference cast for the digital workflow was done on dry cast which may not truly reflect oral condition, and the interdental undercuts of the reference cast was blocked out with wax before alginate impression and this may not be standard clinical practice.

**CONCLUSION**

Suggest using the term ‘superior precision results’ to be more specific to the study findings.

**ACKNOWLEDGEMENT**

Suggest indicating if Berfin Yatmaz is a person or a company.
