# Supplementary material for: In-vitro accuracy of casts for orthodontic purposes obtained by a conventional and by a printer workflow
Source: PLoS One. 2023 Mar 15;18(3):e0282840. doi: 10.1371/journal.pone.0282840 (PMC10016668; doi:10.1371/journal.pone.0282840)
Supplement: Attachment — Submitted filename: 2022.08.20_Plos One.docx [file pone.0282840.s002.docx]

**Dear reviewer**

**Thank you very much for the revision of our manuscript entitled ‘In-vitro accuracy of casts for orthodontic purposes obtained by a conventional and by a printer workflow - Accuracy of conventional and printed casts for orthodontic purposes’.**

**Attached you will find the item-by-item response.**

**Thank you very much for the over-all assessment.**

**Reviewer 1:**

There are fundamental issues with the design that need to be addressed in the writing. The conventional alternative method to introral scanning is to use PVS impression material. The use of alginate impressions was never the accepted standard. The manuscript has to be rewritten to emphasize this. Unfortunately, with the potential conflict of interest, the fact that the design favors intraoral scanners is problematic. If the authors really wanted to "in good faith" test the conventional system they should have gone with PVS, not alginate. Accordingly, the paper should highlight this issue and downgrade any conclusions. Also, conventional impression are typically not poured to fabricate aligners. They are usually scanned in impression form OR scanned for inclusion in a digital workflow. Honestly, I was on the fence about recommending "major rewrite" OR "reject", mainly because of the choices in the design.

On the minor side of notes, the English is acceptable, but could use some editing. The introductions goes a lot into some 3D printing technologies that was not actually used in the study nor is in common clinical use.

**Reviewer #1 expresses several concerns we would like to address. Therefore, we split the paragraph into the single concerns and did an item by item response:**

**1.** The conventional alternative method to intraoral scanning is to use PVS impression material. The use of alginate impressions was never the accepted standard. The manuscript has to be rewritten to emphasize this.

**Comment:** The present study deals with orthodontic casts. To our knowledge, in daily practice alginate impressions are the standard methods to fabricate casts in orthodontics – as well for planning, for working and for documentation. For orthodontic purposes alginate is accurate enough, it is cheap and easy in application. Consequently, it was absolutely appropriate to use alginate as impression material to simulate the conventional workflow in orthodontics.

**2.** Unfortunately, with the potential conflict of interest, the fact that the design favors intraoral scanners is problematic. If the authors really wanted to "in good faith" test the conventional system they should have gone with PVS, not alginate.

**Comment:** As explained above the aim of the present study was to compare the conventional workflow for the fabrication of orthodontic casts, so that we were really in good faith to use alginate. Due to the fact that the scanners on market are suitable for the different indications they are used for example for orthodontic and prosthetic indications. Under these auspices it was correct to test alginate and scanner ‘against’ each other. To simulate orthodontic situations, we used fully dentate full arch reference models without any preparations to simulate the orthodontic set-up. Of course, the entire printer workflow was also tested for prosthetic purposes in another study where we used a reference model that comprised prepared teeth: Of course, in this test set up we used PVS impression material to simulate the standard workflow with the appropriate standard material. This study is already published.

**3.** Also, conventional impression are typically not poured to fabricate aligners. They are usually scanned in impression form OR scanned for inclusion in a digital workflow.

**Comment:** In this point, we disagree, too: Two examples: First example: aligners: If aligners are produced by thermoforming – this is also an absolutele common way of aligner fabrication – a physical cast is necessary. This is also necessary if a digital cast is available that represents the initial situation. Then the desired tooth movements are calculated virtually by an orthodontic planning software. The corresponding casts are printed and the thermoforming method is applied. Second example: Retainer fabrication: For that purpose in most situations physical casts are used as work basis or at least to check the fit of the fabricated retainer.

By the way: The direct scanning of impressions exhibits the shortcoming that undercuts might not always be captured.

**4.** The introductions goes a lot into some 3D printing technologies that was not actually used in the study nor is in common clinical use.

**Comment:** In this point we agree to the reviewer, that we addressed to printer technologies more deeper. However, we decided to do that, because the printer technology is a new and emerging field and it might be advantageous for readers that are not so experienced to get some orientation.

**Reviewer #2: Dear Authors,**

The aim of this study was to evaluate whether conventionally produced casts and printed casts for aligner therapy show comparabel full-arch accuracy. While the topic is fitting to the journal scope, some concerns were raised to publish as a scientific paper. Revise the manuscript by following comments.

**Major points**
**1.** All figures were not visible. Please make sure the link to download figures.

**Comment:** We apologize that that the reviewer could not see the figures. The link to download figures is only available for 7 days. Please feel free to ask for a re-link if the link is no longer valid.

Materials and methods
**2.** "best-fit alignment" is a function of CAD software and black box. Add a brief explanation for this alignment.
**Comment:** Thank you for your advice. We added the following brief explanation:

Revised text: p.8, ll. 187-194

The used method for this automated alignment is a so called iterative closest point algorithm, that projects data points on the triangulated surface of test object toward their closed representation on the reference object. These projections are used to transpose the test object closer to the reference object.to calculate a transformation matrix (consisting of translation and rotation) to match the test object closer to the reference surface. This procedure is done iteratively until no further improvement of the accordance between the two objects could be found. Subsequently, the registered meshes were cut identically in the same plane (Fig 5d).

**3.** Ten times measurement for same specimen is not fit to statistical analysis. The specimens should be different on each measurement.

**Comment:** The conventional and the digital workflow were applied ten times and ten casts each (plaster and printed casts) were produced. Each individual cast was measured to compare it with the reference cast. This corresponds to the standard test set-up of studies investigating the accuracy of intraoral scanners and workflows.

**4.** The Discussion section is short and should be enhanced.

**Comment:** Thank you for your input. We extended the Discussion section by the following passages:

Revised text: p.13, ll. 309-317

The phenomenon of the widening of the arch for [REF vs. IOS] in the molar region could be explained by stitching errors. Due to the fact, that a 3D full arch impression has to be built up by a number of single 3D-images, these single exposures have to be joined together by superimposition/registration of identical structural parts. In general, the more individually structured the scanned surface the more accurate is the registration. A proper registration is the prerequisite for a good full arch accuracy. On the other hand, the scan procedure of the anterior teeth, especially the lower ones, is prone to errors by the stitching algorithms, because they are characterized by flat and steep facets and narrow incisal plateaus (19).

p.14, ll. 345-354

The present study revealed that it was possible to fabricate printed casts with adequate full arch accuracy for orthodontic purposes. The results achieved by the printer-workflow were better than the simulated conventional workflow under in-vitro conditions.

In orthodontics a deviation of < 300 µm for gypsum or printed casts from the reference was determined to be clinically acceptable (8, 22). Compared to casts used in prosthodontics these requirements are less strict. (8, 22). Thresholds of <100 µm mean trueness values are considered as highly accurate and acceptable for the clinical workflow in fixed and implant prosthodontics (8, 22). Based on these findings, the accuracy of the investigated gypsum and printed casts can be considered as clinically acceptable for orthodontic purposes.

p. 15, ll. 358-369

Limitations:

This in-vitro study design differs from the in-vivo procedures in some aspects. For the digital workflow, the cast was scanned in an ideal environment. In a clinical situation, intraoral conditions such as saliva, humidity, patient movement and lack of space may influence the accuracy of intraoral scans (26). Regarding the conventional impressions, interdental undercuts were blocked out with wax to avoid tearing out the interdental spaces which might compromise the comparability to actual clinical procedures (27). Furthermore, alginate impressions are likely to be poured after a certain storage time with varying storage conditions in a clinical scenario. This might result in volume changes and distortion of the impression material and therefore in inaccuracies (27). To minimize the risk of volumetric changes in this study, the impressions were poured directly after a disinfection time of 10 minutes.

**Minor points**
Abstract
**5.** "in-vitro" should be described in Italic font. Make sure all related points and revise them.

**Comment:** Done.

**6.** "xx um (SD+-xx um)" should be simplified to "xx +- xx um".

**Comment:** Done.

Materials and methods
**7.** "a standard tessellation language" should be modified to "a stereolithography".
A later standard tessellation language could be described as STL.
".,." is a typo.

**Comment:** Thank you. Done.

**8.** Some company names appeared many times after the first description.

**Comment:** We have deleted them after citation for the first time.

**9.** "stl" should be modified to "STL".

**Comment:** Done.

**10.** "300.000" might be modified to "300,000".

**Comment:** Thank you very much for this correction. Done.

**11.** MAE might be used as a candidate evaluation criteria.

**Comment:**

Thank you for the thought-provoking remark, that the MAE might be better. There were two main reason to choose the RMSE instead.

The RMSE weights larger single deviations more pronounced than the MAE and consequently is a good indicator to make visible outliers which are disadvantageous for the trueness and precision values. Thus, the RMSE is a more stringent method to identify systems that are prone to outliers. Due to the fact, that we applied a similar test up to other studies (own and other ones in the literature) our results could be compared to them within certain limits. These studies often used the RMSE beside absolute mean deviation.

**Reviewer #3:**

This was an in vitro study to compare conventional and digital/printed models.
**1.** Abstract: I find that there were too much abbreviations that it took me a long time to work out what the abstract was describing. I think the authors should limit the abbreviations and formulas to the content of the manuscript to enable readers to understand what the whole text would be about.

**Comment:** Thank you for that valuable advice. We tried several procedures to enhance the readability and the orientation for the reader.

We tried the complete avoidance of abbreviations using the terms

[reference cast vs. gypsum cast] instead of [REF] – [CON]

[reference vs. DLP printed cast] instead of [REF] - [PRINT]

[reference vs. IOS] instead of [REF] - [IOS]

[IOS vs. DLP printed cast] instead of [IOS] – [PRINT]

This seemed too ‘bulky’ to us. Therefore, we decided to suggest the following compromise:

[REF vs. GYPSUM]; [REF vs. DLP], [REF vs. IOS]

We hope these abbreviations are easier to understand.

**2.** Manuscript
I think the author should consider the clinical importance of their study.

**Comment:** Thank you for this advice. We added a paragraph.

Revised text: p. 14, ll. 345-347

The present study revealed that it was possible to fabricate printed casts with adequate full arch accuracy for orthodontic purposes. The results achieved by the printer-workflow were better than the simulated conventional workflow under in-vitro conditions.

**3.** Clinically acceptable threshold should be explained early in the text. The authors should consider if the study is really about the superiority of the printed models or clinically acceptability?

**Comment:** We introduced the acceptable threshold early in the text. According to the null-hypothesis stated at the end of the introduction the paper focuses on the comparison of the ‘printer’ and the ’conventional’ workflow. The clinical threshold is only mentioned to classify the achieved results. We extended the manuscript by the following passages.

Revised text: p.4, ll. 85.86

Introduction:

For cast accuracy, a clinical acceptable error range of < 100 µm to 500 µm was reported (8).

p. 14, ll. 348-354

Discussion:

In orthodontics a deviation of < 300 µm for gypsum or printed casts from the reference was determined to be clinically acceptable (8, 22). Compared to casts used in prosthodontics these requirements are less strict. (8, 22). Thresholds of <100 µm mean trueness values are considered as highly accurate and acceptable for the clinical workflow in fixed and implant prosthodontics (8, 22). Based on these findings, the accuracy of the investigated gypsum and printed casts can be considered as clinically acceptable for orthodontic purposes. gypsum and printed casts can be considered as clinically acceptable for orthodontic purposes.

p.15, ll. 374-378

Conclusion:

Under in-vitro conditions the print workflow using a DLP printer revealed significantly better trueness values compared to the conventional workflow with respect to absolute mean and mean RMSE results.

Both, the gypsum and the 3D printed cast, produced acceptable accuracy.

**4.** What would be the added value for the more complex digital workflow which may be more costly than conventional, when the latter is already practical.

**Comment:** We added the following passage.

Revised text: p.3, ll. 48-52

Based on the intraoral impressions, the orthodontic planning software can virtually simulate the desired tooth movements. After printing the calculated casts, thermoforming techniques can be performed. Next to simplified model editing, the advantages of the digital workflow are unlimited replica production, less fracture risk and light weight of the produced casts (6).

**5.** Furthermore, if the machines are for industrial, how can the clinicians relate as what's accessible to them are commercial machines?

**Comment:** Sorry for the misunderstanding. Primeprint is a dental printer especially for dental indications such as orthodontic or prosthodontic casts. As the only industrial machine the high resolution scanner was used for accuracy measurements.

**6.** Given that the study is only about DLP printer, it would benefit the reader that a general term use for [PRINT] be more specific to DLP. This is now relevant because other types of 3D printers are so easily accessible that readers would now want to focus which printer the study is about for ease of readership, I would suggest for the authors to minimise using formulas in text and describe their work.

**Comment:** Thank you for this idea. We changed the abbreviations as described above. [PRINT] was modified to [DLP].

**7.** The conclusion should reflect the outcome based on the aims of the study.

**Comment:** The null hypothesis stated was: The null hypothesis stated was that gypsum and printed casts did not differ significantly in comparison to a reference cast at p ≤ 0.05 with respect to accuracy. Furthermore, the differences within the digital workflow were investigated, comparing the digital reference cast file with the intraoral scans and the intraoral scans with the scans of the printed casts.

Therefore, we suggest to arrange the conclusion as follows:

Revised text: p.15, ll. 371-381

**Conclusion**

Within the limits of this study, the following conclusions were drawn:

1.	Under in-vitro conditions the print workflow using a DLP printer revealed significantly better trueness values compared to the conventional workflow with respect to absolute mean and mean RMSE results.

2.	The precision values differed significantly, too.

3.	The results were competitive to the present literature.

4.	Both, the gypsum and the 3D printed cast, produced acceptable accuracy.

5.	Due to contrary deviations in the [REF vs. IOS] and the [IOS vs. DLP] data sets, a compensation of overall trueness deviations is observed.

Reviewer 4: Accuracy of conventional and printed casts for orthodontic purposes

**General**

- This is a well written report.
- The study was thoroughly carried out.

**TITLE**

Adequate

**ABSTRACT**

Adequate

**INTRODUCTION**

Adequate

**MATERIALS AND METHODS**

***Materials & Instruments:***

1. Clarify if the reference cast was scanned and digitized 5 times (line no.89) or 10 times (line no.186).

**Comment:** Thank you. We corrected the inconsistency as follows.

Revised text: p.9, l.218

The high-resolution scans of the reference cast [reference cast] were analyzed for precision, too (n=5).

2. Please describe further details of the standardization of application of artificial saliva (especially in relation to the amount) before taking alginate impression (line no. 104).

**Comment:** We have added the following additional information.

Revised text: p.6, ll.133-135

To ensure a standardized and evenly coating of the cast with the artificial saliva the cast was covered with two defined spray shots by the saliva applicator.

3. As described in Discussion (line no.) which states the moisture sensitive property of alginate, report on the time taken after impression until casting and whether moisture loss from alginate impression was minimized similar to normal clinical situation.

**Comment:** Thank you. The following passage was added.

Revised text: p.6, ll.135-138

As recommended in the manufacturer´s recommendations the impressions were instantly poured with Type IV gypsum (20ml water/100g gypsum) (Rapidur, DENTAURUM GmbH Co. KG) after 10 min disinfection (Kanipur, KANIEDENTA, Dentalmedizinische Erzeugnisse GmbH, Herford, Germany).

**RESULTS**

4. Suggest reporting on precision analysis results of the high-resolution scans of the reference cast [REF] (line no.185).

**Comment:** This result was already reported as follows: p.11, l.273

The precision of the five ATOS scans was 3 µm (SD ± 2 µm).

**DISCUSSION**

5. Discuss the widening of the arch in the molar area with intraoral scanning.

**Comment:** Thank you for the advice. Discussion section was extended as follows.

Revised text: p.13, ll. 309-317

The phenomenon of the widening of the arch for [REF vs. IOS] in the molar region could be explained by stitching errors. Due to the fact, that a 3D full arch impression has to be built up by a number of single 3D-images, these single exposures have to be joined together by superimposition/registration of identical structural parts. In general, the more individually structured the scanned surface the more accurate is the registration. A proper registration is the prerequisite for a good full arch accuracy. On the other hand, the scan procedure of the anterior teeth, especially the lower ones, is prone to errors by the stitching algorithms, because they are characterized by flat and steep facets and narrow incisal plateaus (19).

**LIMITATIONS**

6. Limitations need to be addressed, comment on inherent weakness of in vitro studying, focusing on possible differences in clinical situation. For example, intraoral scanning of the reference cast for the digital workflow was done on dry cast which may not truly reflect oral condition, and the interdental undercuts of the reference cast was blocked out with wax before alginate impression and this may not be standard clinical practice.

**Comment:** Thank you for your input. We included the following paragraph.

Revised text: p.14, ll. 358-369

Limitations:

This in-vitro study design differs from the in-vivo procedures in some aspects. For the digital workflow, the cast was scanned in an ideal environment. In a clinical situation, intraoral conditions such as saliva, humidity, patient movement and lack of space may influence the accuracy of intraoral scans (26). Regarding the conventional impressions, interdental undercuts were blocked out with wax to avoid tearing out the interdental spaces which might compromise the comparability to actual clinical procedures (27). Furthermore, alginate impressions are likely to be poured after a certain storage time with varying storage conditions in a clinical scenario. This might result in volume changes and distortion of the impression material and therefore in inaccuracies (27).

To minimize the risk of volumetric changes in this study, the impressions were poured directly after a disinfection time of 10 minutes.

**CONCLUSION**

7. Suggest using the term ‘superior precision results’ to be more specific to the study findings.

**Comment:** Thank you for your advice. This section was revised as follows.

Revised text: p.15, ll.371-381

1.	Under in-vitro conditions the print workflow using a DLP printer revealed significantly better trueness values compared to the conventional workflow with respect to absolute mean and mean RMSE results.

2.	The precision values differed significantly, too.

3.	The results were competitive to the present literature.

4.	Both, the gypsum and the 3D printed cast, produced acceptable accuracy.

5.	Due to contrary deviations in the [REF vs. IOS] and the [IOS vs. DLP] data sets, a compensation of overall trueness deviations is observed.

**ACKNOWLEDGEMENT**

8. Suggest indicating if Berfin Yatmaz is a person or a company.

**Comment:** Done.

Revised text: p.16, l.384

The study was supported by our colleague Berfin Yatmaz.
