# Supplementary material for: In-vitro accuracy of casts for orthodontic purposes obtained by a conventional and by a printer workflow
Source: PLoS One. 2023 Mar 15;18(3):e0282840. doi: 10.1371/journal.pone.0282840 (PMC10016668; doi:10.1371/journal.pone.0282840)
Supplement: Attachment — Submitted filename: 2022.11.04_Review2.docx [file pone.0282840.s003.docx]

Reviewer #1: Unfortunately, in their response to comments, the authors have decided to dodge some concerns or flat out double down on their shortcomings in the study. There seems to be some confusion in the their arguments against the "conventional workflow". Nowadays, even when physical impressions are made for the sake of aligner fabrication, gypsum casts are seldom used. Yes, in orthodontics we still typically use stone casts for diagnosis, retainer fabrication etc, but who produces a series of gypsum casts for aligner fabrication?? This is obviously the case because it would be pointless to digitally "move" the teeth and create stages then go back to gypsum casts. Once in the digital format, it is typically printed. Regardless, the study is slightly improved compared to last version. I still have some feedback and comments below. With minor tweaks, it will be more acceptable.

**Comment:**

Thank you for pointing out your concerns. Perhaps, our points of view are not too far from each other.

With respect to the digital and the aligner workflow we would like to specify our thoughts:

The starting point of an aligner therapy is either a digital or a conventional impression. In the case of a conventional impression a gypsum cast is usually fabricated. This gypsum cast has to be digitized for the simulation of the tooth movements for aligner therapy. In our study we investigated the accuracy of the conventional workflow, because the first gypsum cast is the origin of the aligner therapy and, therefore, a sufficient accuracy determines the subsequent steps. By the investigation of the digital workflow the following deviations were investigated:

1. reference cast (‘patient’) versus digital cast obtained by an intraoral scanner

2. digital cast versus printed cast (based on the data set of the digital cast)

The digital cast represents the origin of the aligner therapy within a digital workflow comparable to the gypsum cast if the conventional workflow is applied.

For aligner therapy both for the conventional and the digital workflow the virtually simulated tooth movements must be transferred into physical casts by printing to facilitate the fabrication of thermoformed aligners. Therefore, the deviations of the printed casts from their digital casts are important both for the digital and the conventional workflow.

Although we did not simulate any tooth movements the conversion of the digital casts into printed casts can be seen as a representative step both for the digital workflow and the conventional workflow.

The reviewer is right that the accentuation on the aligner aspect in present manuscript is confusing.

Therefore, we would like to suggest some modifications in the manuscript:

**Changes:**

Abstract

Original text:

This *in-vitro* study was designed to investigate whether conventionally produced casts and printed casts for aligner therapy show comparable full-arch accuracy.

Revised text: p.2, ll. 24-25

This *in-vitro* study was designed to investigate whether conventionally produced casts and printed casts for orthodontic purposes show comparable full-arch accuracy. To produce casts, either a conventional impression or a digital data set is needed.

Introduction

Original text:

In orthodontics beside various indications casts are required for aligner fabrication (1, 2).

Revised text: p. 2, ll. 44-45

In orthodontics physical casts are still required for diagnosis and for the fabrication of appliances and retainer therapy (1, 2).

Original text

Based on the intraoral impressions, the orthodontic planning software can virtually simulate the desired tooth movements. After printing the calculated casts, thermoforming techniques can be performed. Next to simplified model editing, the advantages of the digital workflow are unlimited replica production, less fracture risk and light weight of the produced casts (6).

Revised text: p.3, ll. 53-54

~~Based on the intraoral impressions, the orthodontic planning software can virtually simulate the desired tooth movements. After printing the calculated casts, thermoforming techniques can be performed. Next to simplified model editing, t~~The advantages of the digital workflow based on intraoral digital scans are unlimited replica production, less fracture risk and light weight of the produced casts (6).

1) The IOS and alginate impressions were obtained under different circumstances. It would have been very easy for the authors to use artificial saliva and wax block-out for the IOS too. This should be highlighted in the limitations

**Comment:** Thank you for your advice. We extended the limitations as follows to highlight this aspect.

Revised text: p. 17, ll. 402-409

When the conventional impression taking was simulated, the reference cast was wetted with artificial saliva. The artificial saliva was used to reduce the adhesion of the alginate material on the dry surface of the reference cast. Due to the fact that the surface for the optical impression should be free of saliva during that workflow, no artificial saliva was applied. Prior to alginate impression taking, interdental undercuts were blocked out with wax to avoid tearing out the interdental spaces which might compromise the comparability to actual clinical procedures (28). This was not necessary for the simulation of intraoral scanning procedure.

2) The authors need to report the details of the type and properties of the "gypsum used" as different types of dental stones have different properties.

**Comment:** Thank you for this input, the following information was included.

Revised text: p. 6, ll. 144-148

As recommended in the manufacturer´s recommendations the impressions were instantly poured with Type IV gypsum (20ml water/100g gypsum; solidification time ≤ 30 min, setting expansion ≤ 0.15 %, compressive strength ≥ 35 MPa) (Rapidur, DENTAURUM GmbH Co. KG) after 10 min disinfection (Kanipur, KANIEDENTA, Dentalmedizinische Erzeugnisse GmbH, Herford, Germany).

3) DLP printers differ significantly in print quality. The high-end printer and resin used might not be completely comparable to lower-end or more affordable options. This no way a criticism of the study, just a mere comment.

**Comment:** We included this aspect in the discussion as follows:

Revised text: p. 16, ll. 380-382

Primeprint as a printer especially for dental indications applying the DLP technique may differ in print quality from more affordable options due to high-end settings and equipment.

4) Was it not possible for the authors to directly compare DLP vs. Gypsum in table 1.

**Comment:** Thank you for this point but this is an unusual way in studies of this type. The direct comparison between the individual samples is not conclusive because, for example sample 1 from the conventional workflow is not directly related to sample 1 from the digital workflow.

Therefore, only the difference between the mean and median values could be calculated. This should be obvious in the table by comparing the individual values in the rows.

5) In the discussion, line 285, it is not relevant to the study to mention brackets. As stated by the authors, the study is about aligners, which makes brackets irrelevant.
**Comment:** According to the reviewer´s proposal we widened the focus of the indication of orthodontic casts so that the ‘bracket situation’ could remain.

6)The conclusions need some editing. Point 1 and 2 of the conclusions can be merged together.
 Replace the term "conventional" with "alginate-based" OR "alginate/gypsum" in the conclusions.

**Comment:** Thank you for your advice. This section was revised as follows.

Revised text: p. 18, ll. 426-434

Within the limits of this study, the following conclusions were drawn:

1. Under in-vitro conditions the print workflow using a DLP printer revealed significantly better trueness and precision values compared to the alginate/gypsum-based workflow with respect to absolute mean and mean RMSE results.
2. The results were competitive to the present literature.
3. Both, the gypsum and the 3D printed cast, produced acceptable accuracy.
4. Due to contrary deviations in the [REF vs. IOS] and the [IOS vs. DLP] data sets, a compensation of overall trueness deviations is observed.

Reviewer #2: Dear Authors,

The manuscript was mostly revised according to the reviewer's comments. However, all figures were still not visible from the embedded link. The link has been expired. Please put all figures in the main text.

**Comment:** Please excuse any conveniences caused by the unavailable link. We have now added the figures in the manuscript.
